# Comparison of three different therapeutic interventions in the management of knee osteoarthritis: Randomized controlled parallel group pilot trial

**DOI:** 10.1016/j.ocarto.2025.100697

**Published:** 2025-10-17

**Authors:** Vilma Dudonienė, Daumantas Bitinas, Laura Žlibinaitė

**Affiliations:** a Department of Health Promotion and Rehabilitation, Lithuanian Sports University, Sporto 6, Kaunas, LT-44221, Lithuania

**Keywords:** Osteoarthritis, Quality of life, Inpatients, Cryotherapy, Joint mobilization

## Abstract

**Objective:**

The study aimed to compare the effectiveness of different physical interventions on pain intensity, knee function, and quality of life in patients with knee osteoarthritis (OA).

**Methods:**

This pilot trial involved 63 patients (45–55 ​yrs) in a rehabilitation center. Patients were randomly assigned to three groups: therapeutic exercise alone (TE, n ​= ​21), TE and cryotherapy (TE-Cr, n ​= ​21), and TE and joint mobilization (TE-JM, n ​= ​21). The primary outcome was pain intensity, secondary outcomes included knee joint function (WOMAC), muscle strength, knee joint range of motion (ROM), and quality of life (SF-36). Data were collected at baseline and after 18 days of inpatient rehabilitation.

**Results:**

There were no significant between-group differences in the primary outcome at baseline. After 18 days, all intervention groups showed significant improvements (p ​< ​0.05). The TE-JM group reported lower (p ​< ​0.05) pain levels (3.24 ​± ​1.04) compared to the TE-only (4.76 ​± ​0.77) and TE-Cr (4.86 ​± ​0.57) groups. The TE-Cr group had a lower (p ​< ​0.05) SF-36 total score (52.81 ​± ​10.50) than the TE (62.00 ​± ​9.74) and TE-JM (66.62 ​± ​2.87) groups. No significant between-group differences were observed in ROM or muscle strength. The WOMAC total score was lower (p ​< ​0.05) in the TE-JM group (27.3 ​± ​13.9) compared to the TE-Cr group (40.1 ​± ​10.7).

**Conclusion:**

Although all three interventions had beneficial short-term effects, leading to reductions in knee pain and improvements in physical function and quality of life, but no single intervention demonstrated superior effectiveness across all assessed outcomes.

**Trial registration:**

ClinicalTrials.gov, http://www.clinicaltrials.gov, NCT05636059.

## 1 Introduction

Osteoarthritis (OA) is the most common joint disorder worldwide [1], characterized by pain, morning stiffness, limited range of motion (ROM), and muscle weakness [2]. The prevalence of osteoarthritis is increasing [3] and varies widely, ranging from 9.5 ​% to 38.45 ​%, depending on the region of residence [4]. This upward trend in morbidity is also evident in Lithuania, where the prevalence of arthropathies, including osteoarthritis, was 87.5 per 1000 inhabitants in 2017, increased to 92.7 per 1000 in 2018, and reached 97.5 per 1000 in 2019 [5]. Furthermore, the number of prevalent cases increases with age, peaking between 55 and 59 years, and then declines [3].

There is also growing evidence that symptoms of OA can be mitigated through non-pharmacological approaches like patient education, exercise, or weight management [[6], [7], [8]]. Exercise stands as a cornerstone treatment in knee OA [9], with physical interventions showing potential to alleviate pain and enhance ROM [8,10,11]. Physiotherapy regimens, incorporating muscle strengthening and aerobic exercise, are currently regarded as pivotal elements in knee OA management [8]. Furthermore, exercise should be advocated regardless of age, disease severity, functional status, pain levels, or comorbidities [12]. A meta-analysis of 103 trials revealed that the efficacy of exercise varied based on the type of exercise and target outcome; aerobic or mind–body exercises were deemed most beneficial for pain and function improvements, while strengthening and flexibility exercises were effective for multiple outcomes, and mixed exercise was the least effective [13]. In addition to land-based exercises, other therapeutic interventions such as aquatic exercise [9], cryotherapy [14], manual therapy [[15], [16], [17], [18]], and electrotherapy [19] may be recommended for knee OA patients.

Manual techniques such as Joint Mobilization (JM) are hands-on procedures that typically involve the application of rhythmic oscillatory motions of the joint surfaces within the normal joint range [16], aiming to improve the extensibility of contractile tissues and joint movement [17]. Mobilization of an osteoarthritic knee joint immediately produces both local and widespread hypoalgesic effects [18] by enhancing descending pain mechanisms [14], and these effects may also be observed at the one-year follow-up [19]. Several studies [[19], [20], [21]] have reported that joint mobilization, when combined with exercise, provides benefits in managing pain levels, knee ROM, quadriceps muscle strength, and functional level.

In addition to manual therapy techniques and therapeutic exercise, cryotherapy is commonly employed in the management of knee OA. Cryotherapy modalities include ice packs, ice cubes, cold compresses, cold sprays, cold water immersion (cold tubs), ice massage, and cryochambers [14]. Cryochambers are designed for whole-body cryotherapy, in which the entire body, including the head, is exposed to extremely cold temperatures [22]. In the present study, whole-body cryotherapy was selected due to evidence suggesting its effectiveness in reducing pain perception, decreasing the need for analgesic medication, and improving ROM in patients with OA [23]. Additionally, when applied post-exercise, cryotherapy may enhance muscle recovery by attenuating the inflammatory response [24].

Strengthening exercises have been shown to reduce pain and improve physical function and quality of life (QoL) in individuals with knee OA. Consequently, it has been recommended to investigate the potential benefits of adjunctive therapies used in combination with exercise [25]. However, current evidence on the efficacy of combined treatment modalities for OA is limited. Evaluating QoL outcomes may offer healthcare professionals a more comprehensive understanding of the disease and support the development of more effective management strategies [26].

Therefore, the aim of this three-arm parallel-group clinical trial was to evaluate the therapeutic effects of combined interventions, including joint mobilization and cryotherapy, when used alongside therapeutic exercise in patients with knee OA.

## 2 Materials and methods

### 2.1 Ethical approval

Ethical approval was obtained from the University Bioethics Committee (No. MNL-KIN(M)-2021-361). All participants received study details and gave written informed consent. The study adhered to the Declaration of Helsinki and Good Clinical Practice and is registered at ClinicalTrials.gov (ID: NCT05636059).

### 2.2 Study design

In Lithuania, the State Health Insurance Fund covers an 18-day inpatient rehabilitation program for osteoarthritis, involving daily therapeutic interventions (excluding Sundays) in a controlled clinical environment with standardized living and dietary conditions.

This assessor-blinded randomized controlled trial followed a pre-post design over 18 days, comparing three parallel supervised inpatient groups: therapeutic exercise alone (TE group, n ​= ​21), therapeutic exercise plus cryotherapy (TE-Cr group, n ​= ​21), and therapeutic exercise plus joint mobilization (TE-JM group, n ​= ​21). Participants were randomly assigned to one of the three groups using pre-generated, blinded codes and automated allocation (random.org).

### 2.3 Participants

**Fig. 1:**
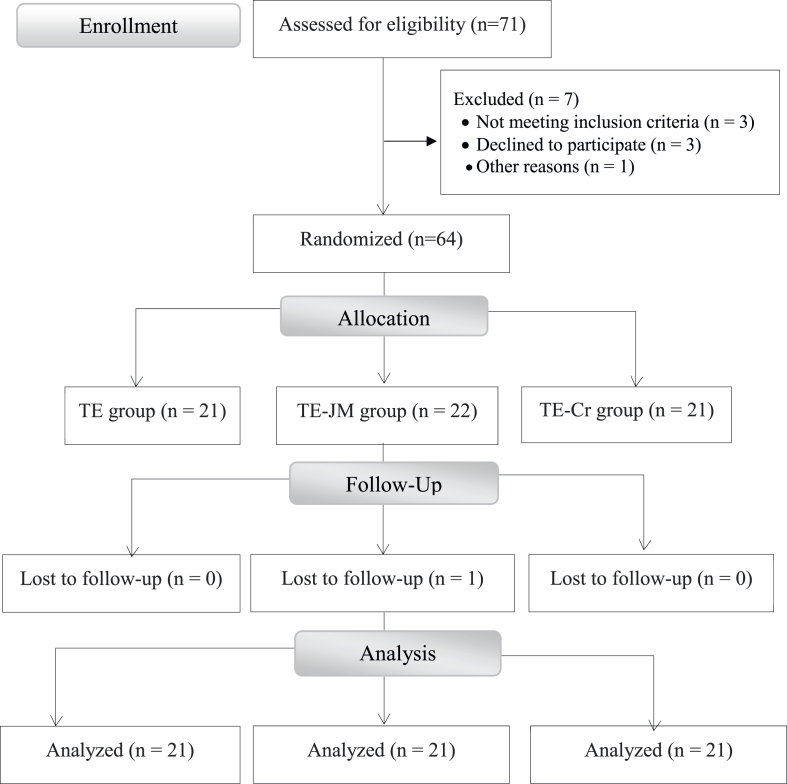
Design of the study (CONSORT flow diagram).

Patients diagnosed with bilateral knee OA by an orthopaedic physician were recruited. Inclusion criteria included Grade II tibiofemoral OA (the Kellgren and Lawrence scale) [27], Visual Analog Scale (VAS) pain score ≥3 in both knees, and age between 45 and 55 years. Exclusion criteria were rheumatoid arthritis, claustrophobia, prior lower limb surgery, neurological disorders, severe cardiovascular dysfunction, cold allergy/intolerance, or impaired lower limb circulation. Based on Whitehead et al. [28], a sample of 60 (20 per group) was deemed sufficient for a pilot study. Of 71 initially screened, 7 were excluded. Sixty-four enrolled, with one dropping out during the intervention phase (Fig. 1).

Table 1 presents the demographic characteristics of the 63 patients who met the inclusion criteria and completed the inpatient rehabilitation program. The mean age was 50.7 ​± ​2.9 years (range 45–55) and 68 ​% were female. There were no significant differences among the three groups in age, sex, body weight, height, or BMI.

**Table 1:** Characteristics of the study participants.

Characteristic	TE group (n ​= ​21) Mean ​± ​SD	TE-Cr group (n ​= ​21) Mean ​± ​SD	TE-JM group (n ​= ​21) Mean ​± ​SD	*P* between groups (one-way ANOVA)
Age (years)	50.9 ​± ​2.8	50.1 ​± ​3.7	51.2 ​± ​2.3	0.282
Women (n, %)	15 (71.4 ​%)	15 (71.4 ​%)	13 (61.9 ​%)	0.873
Height (cm)	168.5 ​± ​8.2	171.5 ​± ​7.0	172.10 ​± ​9.20	0.421
Weight (kg)	82.4 ​± ​12.6	85.4 ​± ​16.6	87.50 ​± ​10.84	0.605
BMI (kg/m^2^)	28.8 ​± ​3.7	29.0 ​± ​4.7	29.70 ​± ​4.50	0.871

Abbreviations: BMI, body mass index; TE, therapeutic exercise; Cr, cryotherapy; JM, joint mobilization.

### 2.4 Outcome measures

#### 2.4.1 Primary

Knee pain intensity was assessed using a 10-cm VAS, with endpoints labelled “no pain” at the left end and “worst pain imaginable” at the right end. Patients marked their current pain level; the distance in cm was recorded as the VAS score: 0 ​= ​no pain, 1–3 ​= ​mild, 4–5 ​= ​moderate, 6–8 ​= ​severe, and 9–10 ​= ​worst imaginable pain [29].

#### 2.4.2 Secondary

*Active knee ROM* was measured from full extension to full flexion using a universal goniometer (Baseline™, UK), following Clarkson's method [30]. Patients lay supine and completed three cycles of maximal knee flexion/extension per leg. Anatomical landmarks included the greater trochanter, lateral femoral condyle, and lateral malleolus. In cases where full knee extension was not achieved, the degree of remaining knee flexion was recorded as a positive angle. The mean of three measurements per leg was used.

*Muscle strength* of knee flexors and extensors was assessed via manual muscle testing using a 0–5 scale: 0 ​= ​no contraction, 5 ​= ​normal strength. Positions varied based on grade: prone/sitting for higher grades (3–5), side-lying for lower (0–2) [30].

*Health-related QoL* was assessed using a life quality assessment questionnaire (SF-36v2® Health Survey Standard, Lithuania). The survey comprised 36 questions reflecting eight life domains: physical functioning, restriction of activity due to physical problems, pain, general health assessment, energy levels and vitality, social functioning, restriction of activity due to emotional disorders, and emotional state. Responses to these questions were scored in each section from 0 to 100 using a calculation algorithm. A higher score indicated better health-related quality of life [31].

*The functional status of the knee* was assessed using the Western Ontario and McMaster Universities Osteoarthritis Index (WOMAC), which comprises three subscales: pain (5 questions), stiffness (2 questions), and functional status (17 questions), totalling 24 items. Each question was scored between 1 and 4, and the total score was calculated for each subscale. Higher WOMAC scores indicated worse self-perceived pain and function [32].

All assessments were conducted at baseline (Day 1) and at discharge (the last day of an 18-day inpatient rehabilitation), in the morning before the intervention started. First the participants rated their current knee pain intensity and, with the assistance of a rehabilitation doctor, completed the questionnaires; ROM and muscle strength were then measured. All measurements were repeated after rehabilitation in the same order. The assessor was blinded to the patients, and all outcomes were assessed by the same person. Adherence to the intervention was high (98 ​%), with only one dropout.

### 2.5 Interventions

Study participants received either TE alone, TE plus Cryotherapy (Cr), or TE plus JM treatments at the inpatient physical medicine and rehabilitation center. The number of sessions varied based on clinical recommendations (Supplementary material, Table S1).

Three experienced physiotherapists led the interventions: one supervised TE for all groups, one performed cryotherapy, and a third, trained in manual therapy, conducted joint mobilization. All therapists were blinded to patients’ assessment results.

Participants were instructed not to take analgesics or nonsteroidal anti-inflammatory drugs to avoid the potential analgesic effects of these medications throughout the study period.

#### 2.5.1 Therapeutic exercise (TE) only group

The TE program consisted of aerobic, strengthening, and stretching exercises to improve function and reduce symptoms in knee osteoarthritis. Participants cycled on a stationary bicycle for up to 10 ​min at a self-selected pace, aiming to increase cardiovascular endurance and joint mobility. The strengthening component included both isometric and isotonic exercises targeting the quadriceps, hip flexors, extensors, adductors, and abductors. Isometric exercises involved maximal voluntary contractions held for 10 ​s, with 10 repetitions per muscle group. Isotonic exercises were performed through a full ROM against resistance, also with 10 repetitions per exercise. Static stretching exercises focused on the gastrocnemius-soleus complex and hamstring muscles, with each stretch held for 30 ​s and repeated three times per muscle group to enhance flexibility and reduce stiffness. Each session lasted approximately 30 ​min and was performed twice daily under supervision or with guided instruction. The TE program closely followed the protocol described by Kaya Mutlu et al. [19]. In total, participants completed 32 ​TE sessions over the course of the intervention.

#### 2.5.2 Therapeutic exercise and cryotherapy (TE-Cr) group

Participants in the TE-Cr group received the same TE program as the TE-only group, with the addition of whole-body cryotherapy in Cryochambers [14]. The cryochamber (JUKA, 0401, produced in Poland, 2017, registration number ED 601147480001) had two compartments with temperatures of −60 ​°C and −140 ​°C. Cryotherapy treatment involved exposing the whole body, including the head, to extremely cold dry air for 2 ​min. Cryotherapy sessions were conducted two to three times per week, following TE sessions. While in the cryochamber, participants wore a bathing suit, gloves, cap, socks, and shoes and were advised to pace rhythmically and move their fingers to avoid stiffness. The TE were performed once per day, every consecutive working day. Patients in this group received 24 sessions (16 ​TE and 8 Cr).

#### 2.5.3 Therapeutic exercise and joint mobilization (TE-JM) group

Patients in the TE-JM group received the same exercise program as those in the TE-only group, with the additional inclusion of knee joint mobilization [33]. Therapeutic exercises were performed first, followed by mobilization. Joint mobilization was conducted passively and included posterior and anterior tibial glides and patellar glides in all directions (Supplementary material, Fig. S1), applied at a rate of two to three oscillations (grade I or II) per second for 1–2 ​min. Movements in each direction were repeated three to six times. All glides were performed with the patient in a supine position. Patients in this group received 24 sessions (16 ​TE and 8 JM).

### 2.6 Statistical analysis

Most of the data were found to be normally distributed using the Shapiro-Wilk test. However, for some data that deviated from normality, parametric statistics were still applied, as analyses of variance (ANOVA) are robust to violations [34]. Parametric statistics was used to analyze the SF-36 and WOMAC scales. Although these Likert-based questionnaires collect ordinal data, Norman [35] demonstrated that parametric statistical methods can yield valid conclusions with such data.

One-way ANOVAs were used to assess differences between groups at baseline. Two-way ANOVAs (Group x Test) with repeated measures on the Test Factor were employed to evaluate differences in VAS scores, WOMAC scores, SF-36 scores, ROM, and muscle strength. When statistical significance was observed, Bonferroni post-hoc analyses were performed to identify the actual differences. The level of significance was set at P ​< ​0.05. Effect sizes were reported using partial eta squared for ANOVAs and Cohen's *d* for post-hoc pairwise comparisons. Effect sizes were interpreted as follows: 0.2 for small, 0.5 for medium, and 0.8 for large. All statistical analyses were conducted using IBM SPSS Statistics (version 26.0, IBM Corp., Armonk, NY, USA). Values are reported as means and standard deviations.

## 3 Results

### 3.1 Primary outcome

#### 3.1.1 Pain

The data on pain intensity measured using VAS scale are presented in Table 2. There were no statistical differences between groups in pain intensity according to one-way ANOVA test pre-interventions, F(1, 61) ​= ​0.14, p ​> ​0.05, ƞ2p ​= ​0.00. A two-way ANOVA (Group x Test) with repeated measures on the Test factor revealed a Group ​× ​Test interaction, F(2, 60) ​= ​17.15, p ​< ​0.001, ƞ2p ​= ​0.36. A Bonferroni post hoc analysis revealed that at the post-test, the TE-JM group's pain rating was lower (3.24 ​± ​1.04) compared to the TE group (4.76 ​± ​0.77; p ​< ​0.05, Cohen's *d* ​= ​1.43) and the TE-Cr group (4.86 ​± ​0.57; p ​< ​0.05, Cohen's *d* ​= ​1.52), there were no statistical differences between the TE and the TE-Cr groups. There was also a Test effect, F(1, 60) ​= ​165.93, p ​< ​0.001, ƞ2p ​= ​0.73, as pain ratings were lower in the post-test compared to the pre-test. There was also a Group effect, F(2, 60) ​= ​4.96, p ​< ​0.05, ƞ2p ​= ​0.14.

**Table 2:** Comparison of pain intensity within and between groups.

Outcome	Group	Pre Mean ​± ​SD	Post Mean ​± ​SD	P within groups	Cohen's *d*	*P* between groups
Pain (VAS, cm**)**	TE	6.1 ​± ​1.0	4.8 ​± ​0.8	0.000	*1.45*	
	TE-Cr	5.8 ​± ​1.3	4.9 ​± ​0.6	0.003	*0.88*	0.01
	TE-JM	6.0 ​± ​1.4	3.2 ​± ​1.0	0.000	*2.26*	TE-JM^a^

Abbreviations: TE, therapeutic exercise; Cr, cryotherapy; JM, joint mobilization; VAS, visual analogue scale; SD, standard deviation.

a p ​< ​0.05, different from TE-Cr and TE groups.

### 3.2 Secondary outcomes

The data on QoL assessed using SF-36 questionnaire are presented in Table 3. There were no statistical differences between groups in SF-36 total score according to one-way ANOVA test comparison pre-interventions (F(1, 61) ​= ​1.01, p ​> ​0.05, ƞ2p ​= ​0.02). A two-way ANOVA (Group x Test) with repeated measures on the Test factor revealed a Group ​× ​Test interaction, F(2, 60) ​= ​176.13, p ​< ​0.001, ƞ2p ​= ​0.36. A Bonferroni post hoc analysis revealed that at the post-test, the TE-Cr group's SF-36 total score was lower (52.81 ​± ​10.50) compared to the TE group (62.00 ​± ​9.74; p ​< ​0.05, Cohen's *d* ​= ​0.91) and the TE-JM group (66.62 ​± ​2.87; p ​< ​0.001, Cohen's *d* ​= ​1.79). There were no differences between the TE-JM and the TE groups. There was also a Test effect, F(1, 60) ​= ​105.49, p ​< ​0.001, ƞ2p ​= ​0.64. There was also a Group effect, F(2, 60) ​= ​10.24, p ​< ​0.001, ƞ2p ​= ​0.26.

**Table 3:** Comparison of SF-36 questionnaire and its separate domains within and between groups.

SF-36 domains	Group	Pre Mean ​± ​SD	Post Mean ​± ​SD	P within groups	Cohen's *d*	*P* between groups
Physical functioning	TE	58.8 ​± ​20.1	70.7 ​± ​19.2	0.001	*0.61*	0.871
	TE-Cr	56.4 ​± ​20.3	68.3 ​± ​17.6	0.003	*0.63*	
	TE-JM	52.6 ​± ​19.4	72.1 ​± ​12.5	0.000	*1.19*	
Role-physical	TE	73.8 ​± ​39.9	73.8 ​± ​39.9	1.000	*-*	0.005 TE-JM^a^
	TE-Cr	48.8 ​± ​36.6	51.2 ​± ​39.1	0.157	*0.06*	
	TE-JM	75.0 ​± ​29.6	92.9 ​± ​16.1	0.015	*0.75*	
Bodily pain	TE	40.9 ​± ​14.8	43.5 ​± ​15.4	0.025	*0.17*	0.061
	TE-Cr	38.2 ​± ​18.0	42.4 ​± ​18.2	0.005	*0.23*	
	TE-JM	19.9 ​± ​10.8	43.0 ​± ​18.7	0.000	*1.51*	
General health	TE	48.8 ​± ​9.7	51.4 ​± ​8.4	0.001	*0.29*	0.009 TE-JM^a^
	TE-Cr	47.4 ​± ​10.0	54.3 ​± ​9.8	0.000	*0.70*	
	TE-JM	56.0 ​± ​8.6	60.5 ​± ​9.1	0.000	*0.51*	
Energy and vitality	TE	61.9 ​± ​6.4	62.4 ​± ​6.8	0.414	*0.08*	0.000 TE-Cr^c^
	TE-Cr	46.9 ​± ​17.5	54.6 ​± ​14.7	0.001	*0.81*	
	TE-JM	61.0 ​± ​4.9	64.5 ​± ​3.5	0.005	*0.50*	
Social functioning	TE	45.1 ​± ​13.0	45.1 ​± ​13.0	1.000	*-*	0.071
	TE-Cr	38.8 ​± ​5.6	41.9 ​± ​4.4	0.014	*0.61*	
	TE-JM	43.1 ​± ​5.6	48.7 ​± ​5.6	0.005	*0.99*	
Role-emotional	TE	87.2 ​± ​30.7	87.2 ​± ​30.7	1.000	*-*	0.001 TE-Cr^c^
	TE-Cr	53.8 ​± ​42.8	53.8 ​± ​42.8	1.000	*-*	
	TE-JM	83.9 ​± ​22.9	90.3 ​± ​15.7	0.063	*0.33*	
Emotional state	TE	61.7 ​± ​7.2	62.9 ​± ​6.7	0.063	*0.17*	0.034 TE-Cr^b^
	TE-Cr	54.0 ​± ​7.9	59.6 ​± ​7.0	0.001	*0.75*	
	TE-JM	57.9 ​± ​6.8	62.1 ​± ​6.0	0.000	*0.66*	
SF-36 total score	TE	59.6 ​± ​10.5	62.0 ​± ​9.7	0.000	*0.24*	0.000 TE-Cr^c^
	TE-Cr	47.9 ​± ​11.3	52.8 ​± ​10.5	0.000	*0.45*	
	TE-JM	56.2 ​± ​7.4	66.6 ​± ​2.9	0.000	*1.85*	

Abbreviations: TE, therapeutic exercise; Cr, cryotherapy; JM, joint mobilization; SD, standard deviation.

a p ​< ​0.05 different from TE-Cr.

b p ​< ​0.05 different from TE group.

c p ​< ​0.05 different from TE-JM and TE groups.

Significant within-group improvements were observed in the TE-JM group with the largest effect sizes (Cohen's *d = 1.43* for Bodily Pain and *d = 1.75* for SF-36 total score). The TE-Cr group showed improvements in multiple domains, although the effect sizes were generally smaller than those observed in the TE-JM group. In contrast, the TE-only group showed statistically significant changes in fewer domains, with mostly small effect sizes and no change in Role-Physical, Social Functioning, or Role-Emotional.

#### 3.2.1 Functional status of the knee

The data on functional status of the knee assessed using WOMAC index are presented in Table 4. There were no statistical differences between groups in WOMAC total score according to one-way ANOVA test comparison pre-interventions (F(1, 61) ​= ​0.54, p ​> ​0.05, ƞ2p ​= ​0.01). A two-way ANOVA (Group x Test) with repeated measures on the Test factor revealed a Group ​× ​Test interaction, F(2, 60) ​= ​6.70, p ​< ​0.05, ƞ2p ​= ​0.18. A Bonferroni post hoc analysis revealed that at the post-test, the TE-JM group's WOMAC total scores were lower (27.29 ​± ​13.92) compared to the TE-Cr group (40.10 ​± ​10.74; p ​< ​0.05, Cohen's *d* ​= ​0.45). There were no statistical differences between the TE and the TE-Cr groups. There was also a Test effect, F(1, 60) ​= ​104.40, p ​< ​0.001, ƞ2p ​= ​0.64. There was also a Group effect, F(2, 60) ​= ​4.67, p ​< ​0.05, ƞ2p ​= ​0.14.

**Table 4:** Comparison of WOMAC scale and its separate domains within and between groups.

Outcome	Group	Pre Mean ​± ​SD	Post Mean ​± ​SD	P within groups	Cohen's *d*	*P* between groups
WOMAC - pain	TE	35.7 ​± ​16.4	30.7 ​± ​14.1	0.002	*0.33*	0.622
	TE-Cr	36.0 ​± ​13.8	30.5 ​± ​13.7	0.000	*0.40*	
	TE-JM	38.1 ​± ​13.3	21.0 ​± ​15.3	0.000	*1.19*	
WOMAC - stiffness	TE	34.8 ​± ​10.4	33.5 ​± ​12.1	0.180	*0.12*	0.606
	TE-Cr	50.3 ​± ​14.7	25.0 ​± ​21.6	0.000	*1.42*	
	TE-JM	40.1 ​± ​16.5	36.6 ​± ​14.2	0.023	*0.23*	
WOMAC – physical function	TE	40.1 ​± ​9.5	36.5 ​± ​10.8	0.000	*0.35*	0.012 TE-Cr^b^
	TE-Cr	51.0 ​± ​16.3	45.3 ​± ​9.4	0.003	*0.43*	
	TE-JM	38.1 ​± ​13.3	27.9 ​± ​14.6	0.000	*0.73*	
WOMAC – total	TE	39.0 ​± ​9.8	34.9 ​± ​10.5	0.000	*0.40*	0.013 TE-JM^a^
	TE-Cr	45.0 ​± ​11.1	40.1 ​± ​10.7	0.000	*0.45*	
	TE-JM	36.2 ​± ​13.4	27.3 ​± ​13.9	0.000	*0.65*	

Abbreviations: TE, therapeutic exercise; Cr, cryotherapy; JM, joint mobilization; SD, standard deviation.

a p ​< ​0.05 different from TE-Cr group.

b p ​< ​0.05 different from TE-JM and TE groups.

Significant within-group improvements were observed in the TE-JM group, with the largest effect size recorded in the pain domain (Cohen's *d* ​= ​1.12). The TE-Cr group also demonstrated improvements across several domains, although the effect sizes were generally smaller than those in the TE-JM group. In contrast, the TE-only group showed statistically significant improvements in all domains except stiffness, however, the corresponding effect sizes were small to moderate.

#### 3.2.2 Range of motion

The data on ROM and muscle strength are presented in Table 5. A two-way ANOVA (Group ​× ​Test), with repeated measures on the Test factor, revealed a significant main effect of Test for all ROM measures. Significant improvements were observed from pre-to post-test in right knee flexion (F(1, 60) ​= ​103.06, p ​< ​0.001, ƞ2p ​= ​0.63), left knee flexion (F(1, 60) ​= ​105.36, p ​< ​0.001, ƞ2p ​= ​0.64), right knee extension (F(1, 60) ​= ​22.15, p ​< ​0.001, ƞ2p ​= ​0.27) and left knee extension (F(1, 60) ​= ​24.25, p ​< ​0.001, ƞ2p ​= ​0.29). However, no significant Group ​× ​Test interaction or between-group differences in ROM were detected. Cohen's effect sizes ranged from small to medium.

**Table 5:** Comparison of range of motion and muscle strength within and between groups.

Outcome	Group	Pre Mean ​± ​SD	Post Mean ​± ​SD	P within groups	Cohen's *d*	*P* between groups
**Range of motion (°)**						
Right knee flexion	TE	119.4 ​± ​7.5	123.3 ​± ​7.6	0.000	*0.52*	0.188
	TE-Cr	123.7 ​± ​9.6	127.8 ​± ​7.0	0.000	*0.49*	
	TE-JM	121.4 ​± ​7.9	125.2 ​± ​7.1	0.000	*0.44*	
Right knee extension	TE	1.5 ​± ​1.8	0.4 ​± ​0.8	0.027	*0.72*	0.119
	TE-Cr	1.1 ​± ​1.2	0.5 ​± ​0.9	0.008	*0.57*	
	TE-JM	0.3 ​± ​0.7	0.1 ​± ​0.3	0.014	*0.39*	
Left knee flexion	TE	117.7 ​± ​10.1	123.8 ​± ​8.4	0.000	*0.61*	0.108
	TE-Cr	122.0 ​± ​8.3	127.9 ​± ​5.6	0.000	*0.75*	
	TE-JM	115.9 ​± ​11.6	123.6 ​± ​6.8	0.001	*0.78*	
Left knee extension	TE	1.8 ​± ​2.3	0.8 ​± ​1.3	0.011	*0.54*	0.104
	TE-Cr	1.1 ​± ​1.9	0.3 ​± ​0.6	0.017	*0.57*	
	TE-JM	0.7 ​± ​1.0	0.2 ​± ​0.4	0.015	*0.67*	
**Muscle strength (grades)**						
Right knee flexion	TE	4.2 ​± ​0.4	4.6 ​± ​0.5	0.005	*0.46*	0.643
	TE-Cr	4.1 ​± ​0.3	4.7 ​± ​0.5	0.000	*0.52*	
	TE-JM	4.2 ​± ​0.4	4.8 ​± ​0.4	0.000	*0.57*	
Right knee extension	TE	4.4 ​± ​0.5	4.7 ​± ​0.5	0.008	*0.26*	0.088
	TE-Cr	4.2 ​± ​0.4	4.7 ​± ​0.5	0.001	*0.47*	
	TE-JM	4.8 ​± ​0.4	4.9 ​± ​0.3	0.083	*0.18*	
Left knee flexion	TE	4.2 ​± ​0.4	4.7 ​± ​0.5	0.002	*0.33*	0.593
	TE-Cr	4.0 ​± ​0.0	4.8 ​± ​0.4	0.000	*0.63*	
	TE-JM	4.3 ​± ​0.6	4.7 ​± ​0.5	0.003	*0.39*	
Left knee extension	TE	4.4 ​± ​0.5	4.8 ​± ​0.5	0.005	*0.25*	0.099
	TE-Cr	4.2 ​± ​0.4	4.7 ​± ​0.5	0.002	*0.41*	
	TE-JM	4.7 ​± ​0.5	4.8 ​± ​0.4	0.083	*0.19*	

Abbreviations: TE, therapeutic exercise; Cr, cryotherapy; JM, joint mobilization; SD, standard deviation.

#### 3.2.3 Muscle strength

A two-way ANOVA (Group ​× ​Test) with repeated measures on the Test factor revealed a significant Test effect for all muscle strength measures, with right knee flexion (F(1, 60) ​= ​74.10, p ​< ​0.001, ƞ2p ​= ​0.55), right knee extension (F(1, 60) ​= ​33.66, p ​< ​0.001, ƞ2p ​= ​0.36), left knee flexion (F(1, 60) ​= ​82.22, p ​< ​0.001, ƞ2p ​= ​0.56), and left knee extension (F(1, 60) ​= ​32.91, p ​< ​0.001, ƞ2p ​= ​0.35) demonstrating significantly higher values in the post-test compared to the pre-test. Additionally, a significant Group ​× ​Test interaction was found (F(2, 60) ​= ​3.66, p ​= ​0.03, ƞ2p ​= ​0.11); however, post hoc analysis indicated no significant differences between groups at the post-test.

## 4 Discussion

The primary objective of this study was to evaluate whether the addition of cryotherapy and joint mobilization to standard physiotherapy treatment, which includes physical exercises, would improve treatment outcomes for patients with knee OA in an inpatient rehabilitation setting.

All three interventions applied in this study, therapeutic exercise only (TE), therapeutic exercise combined with joint mobilization (TE-JM), and therapeutic exercise combined with cryotherapy (TE-Cr), resulted in significant improvements in symptoms, physical function, and QoL in patients with early-stage knee OA. Patients tolerated all procedures well, and no adverse side effects were reported, although some patients found the passive manual movements somewhat uncomfortable.

Patient education, physical exercise, and weight loss for overweight or obese individuals are considered the first-line treatment approach for knee osteoarthritis (OA) [14]. A majority of the study participants (68.3 ​%) were women with an elevated BMI, which supports existing evidence that being female and having a higher BMI significantly increase the risk of developing knee OA [36].

Pain is a primary outcome in OA research and is commonly assessed using VAS and the WOMAC index [13]. In the present study, all three interventions led to significant reductions in knee pain according to both VAS and WOMAC scores. However, the TE-JM group showed a significantly greater reduction in pain based on VAS scores, while no difference was observed compared to TE only group on WOMAC pain domain. A study of Kaya Mutlu et al. [19] demonstrated that both active and passive joint mobilization effectively reduce pain, with the analgesic effect potentially lasting up to one year. By improving joint mobility, joint mobilization helps reduce mechanical stress on the joint and associated pain. When combined with therapeutic exercise, which focuses on strengthening the muscles around the joint and improving overall function, the two interventions complement each other in restoring optimal joint mechanics. In the intention-to-treat analysis, all intervention groups showed improvement, but only the TE-JM group achieved a clinically significant reduction of 1.75 ​cm on the VAS scale [14]. The findings of this study provide indirect support for the potential benefits of joint mobilization. A systematic review by Tsokanos et al. [15] concluded that manual therapy, including joint mobilization, positively affects functionality in knee OA patients. Courtney et al. [16] found that joint mobilization interventions increase pain thresholds and decrease pain levels. Furthermore, evidence suggests that joint mobilization may modulate pain by enhancing descending pain inhibitory mechanisms in patients with painful knee OA [16].

QoL assessments are essential in the management of knee osteoarthritis (OA) [26]. In this study, the total SF-36 score increased significantly within all groups. Although no statistically significant differences were found between the TE-only and TE-JM groups post-intervention, the TE-Cr group demonstrated significantly lower QoL compared to the other two groups. As the SF-36 is a self-reported and subjective questionnaire, its results can be influenced by the respondent's current emotional state. Therefore, it may not always be sensitive enough to detect subtle differences between similar interventions, especially over a short intervention period.

In this study, knee functional status, as measured by the WOMAC questionnaire, improved significantly within all groups. However, no statistically significant differences were found between groups in the WOMAC pain and stiffness subscales. Interestingly, the TE-Cr intervention was the most effective in improving physical function, while the TE-JM group showed the greatest improvement in the WOMAC total score. Sit et al. [37] reported that patellar mobilization therapy can potentially reduce pain and improve function and QoL in patients with knee OA. Although García et al. [38] reported that cryotherapy reduces inflammatory processes, potentially explaining its effect on stiffness, such an effect was not observed in the present study. Furthermore, some studies have reported insufficient evidence regarding the long-term effectiveness of cryotherapy in relieving pain and improving physical function in individuals with knee OA [14].

Nonoperative therapeutic interventions for knee OA commonly aim to enhance quadriceps strength and improve knee ROM [12]. In the present study, all three intervention groups demonstrated significant within-group improvements in knee flexion and extension ROM, with small to medium effect sizes. Structured exercise programs targeting lower extremity muscle strengthening are widely recommended for patients with knee OA and have been shown to effectively reduce pain and improve functional status [8,12,13,39]. No significant between-group differences were found in ROM or muscle strength after the interventions. Since all participants underwent the same therapeutic exercise program, the addition of joint mobilization or cryotherapy did not appear to confer additional benefits in these outcomes.

It is important to consider the age range of patients when interpreting study results. The age range of participants, 45–55 years, is relatively young for knee OA [40]. Losina et al. [39] estimated that the diagnosis of symptomatic knee OA occurs early in the life course (median age 55 years). This suggests that public health professionals should introduce prevention strategies relatively early in the life course, and those prevention strategies should be aimed at reducing obesity [41,39], preventing sport injuries [41] and involving people in physical activity [39].

This clinical trial has several limitations. These include a relatively small sample size, a short intervention duration of only 18 days, and variability in the number of procedures performed among participants. Additionally, more specific and instrumented assessment methods should be employed to enhance the accuracy of outcome measurements. However, all participants were at the same disease stage, the age range was narrow, and the interventions were implemented in a clinical setting under the supervision of experienced physiotherapists. The groups were comparable in terms of gender distribution, which supports internal validity. While the findings may not be generalizable to older populations or patients with more advanced stages of knee OA, they nonetheless provide valuable insights into early-stage management strategies. Future research should address the current limitations by including larger sample sizes and investigating the long-term effects of these interventions. The incorporation of biochemical markers, such as inflammatory cytokines, would also be beneficial for understanding the biological mechanisms underlying symptom changes and treatment efficacy.

## 5 Conclusions

All three interventions, therapeutic exercise alone, therapeutic exercise combined with joint mobilization, and therapeutic exercise combined with cryotherapy, resulted in significant short-term improvements in pain, physical function, and QoL in patients with early-stage knee osteoarthritis. However, no single intervention proved to be consistently superior across all evaluated outcomes.

## Author contributions

Conceptualization, V.D., D.B. and L.Ž.; data collection, D.B.; formal analysis, D.B. and L.Ž.; investigation, D.B.; methodology, V.D. and L.Ž.; project administration, V.D.; software, L.Ž.; validation, V.D., L.Ž. and D.B.; visualization, V.D.; supervision, L.Ž.; writing—original draft, V.D and L.Ž.; writing—review & editing, V.D. and L.Ž. All authors have read and agreed to the final version of the manuscript to be published.

## Institutional review board statement

The study was approved by the LSU Bioethics Committee (Approval No. MNL-KIN(M)-2021-361) and registered at ClinicalTrials.gov (Identifier: NCT05636059). It was conducted in accordance with the ethical principles of the Declaration of Helsinki and Good Clinical Practice guidelines.

## Funding

This research received no external funding.

## Conflicts of interest

The authors declare that they have no conflict of interest.
