# Supplementary material for: Comparison of three different therapeutic interventions in the management of knee osteoarthritis: Randomized controlled parallel group pilot trial
Source: Osteoarthr Cartil Open. 2025 Oct 17;7(4):100697. doi: 10.1016/j.ocarto.2025.100697 (PMC12589867; doi:10.1016/j.ocarto.2025.100697)
Supplement: Multimedia component 1 [file mmc1.docx]

**Table S1.** Description of therapeutic interventions

| Intervention  group | Frequency | Duration of one  session | Number of interventions |
| --- | --- | --- | --- |
| TE | 2 times/day | 30 min | 32 TE |
| TE-Cr | TE – 1 time/day;  Cr – 2-3 times/week | TE – 30 min;  Cr – cryochamber for 2 min, first chamber -60°C, second -140°C | 16 TE plus 8 Cr |
| TE-JM | TE – 1 time/day;  JM – 2-3 times/week | TE – 30 min;  JM – 10-15 min | 16 TE plus 8 JM |

Abbreviations: TE - therapeutic exercise; Cr - cryotherapy; JM - joint mobilization

A
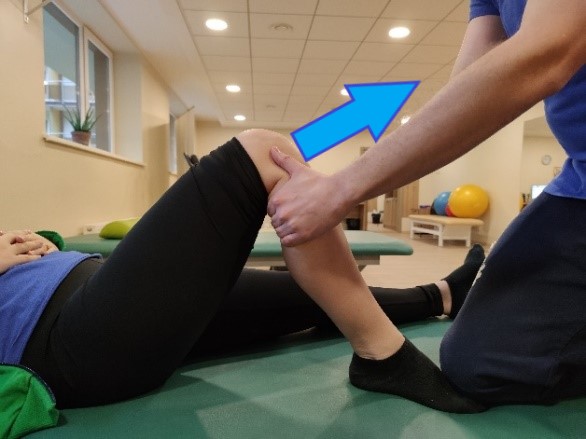
 B
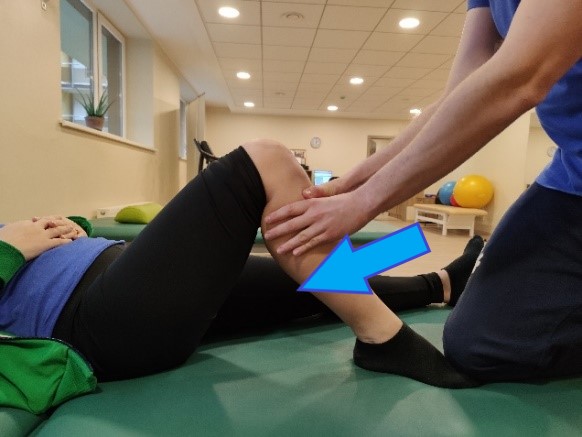


C
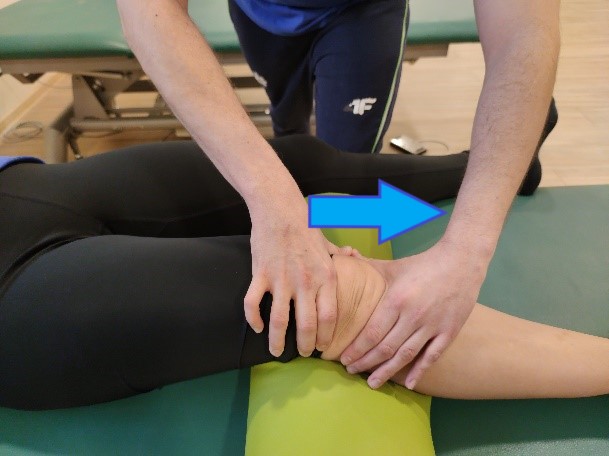
 D
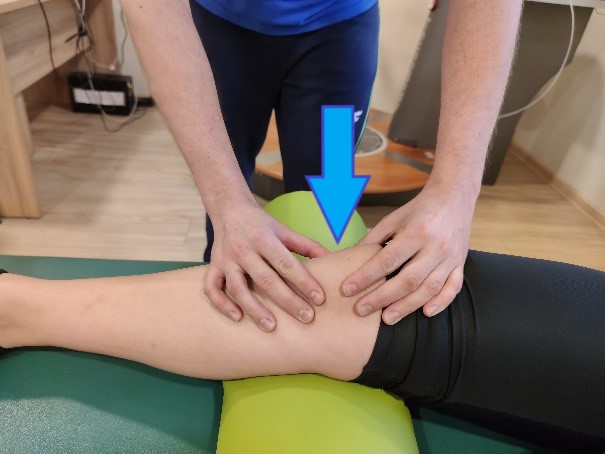


E
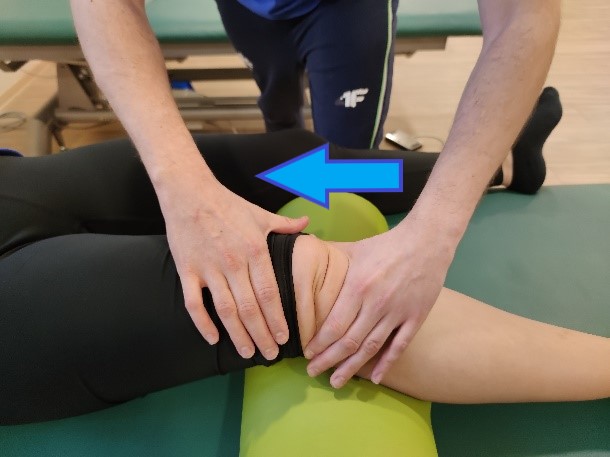
 F
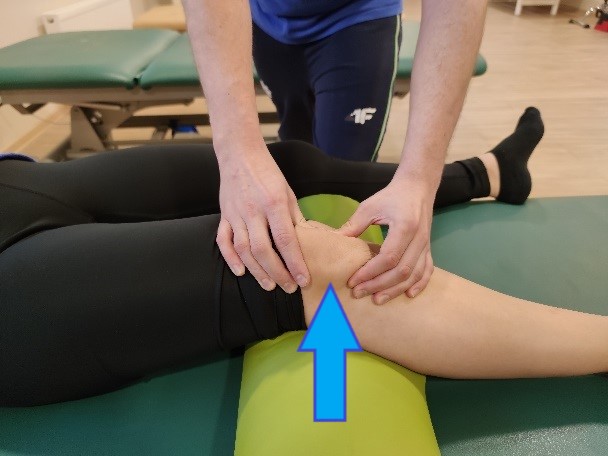


**Figure S1**. Anterior (A) and Posterior (B) tibial glide technique; Passive patellofemoral joint mobilization: C) the inferior glide technique; D) the lateral–medial glide technique; E) the superior glide technique; F) the medial–lateral glide technique


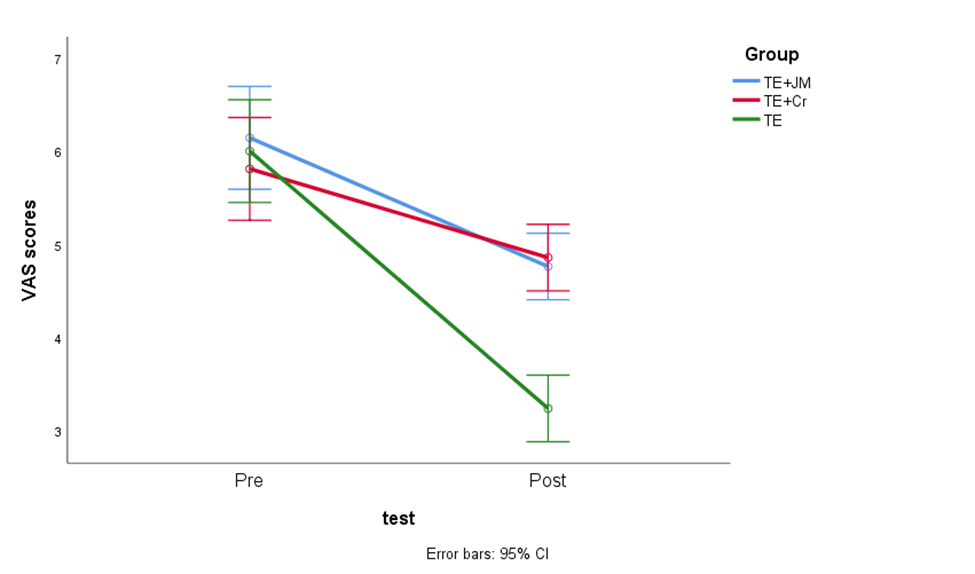


**Figure S2.** The Group x Test interaction in VAS scores. Error bars represent 95% CI2


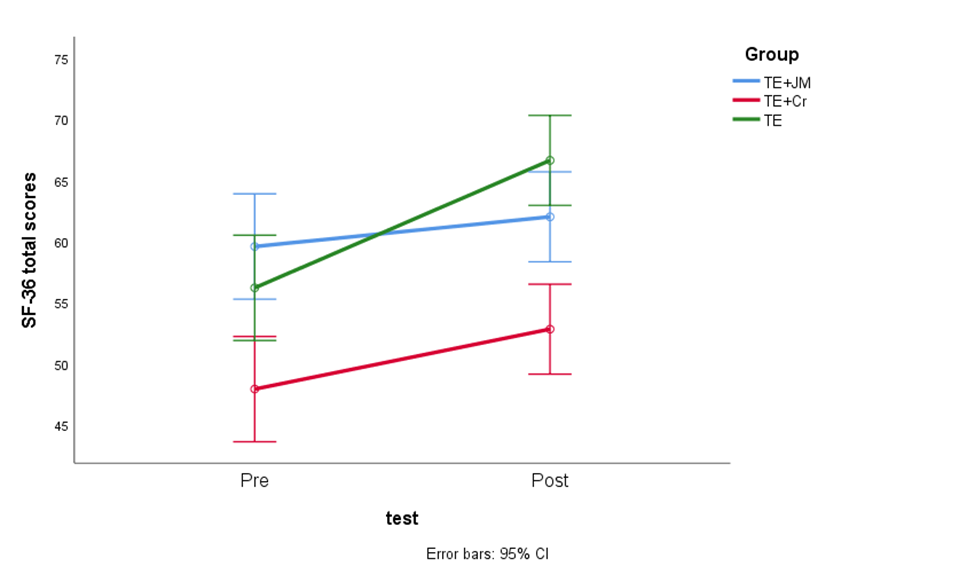


**Figure S3.** The Group x Test interaction in SF-36 total scores. Error bars represent 95% CI


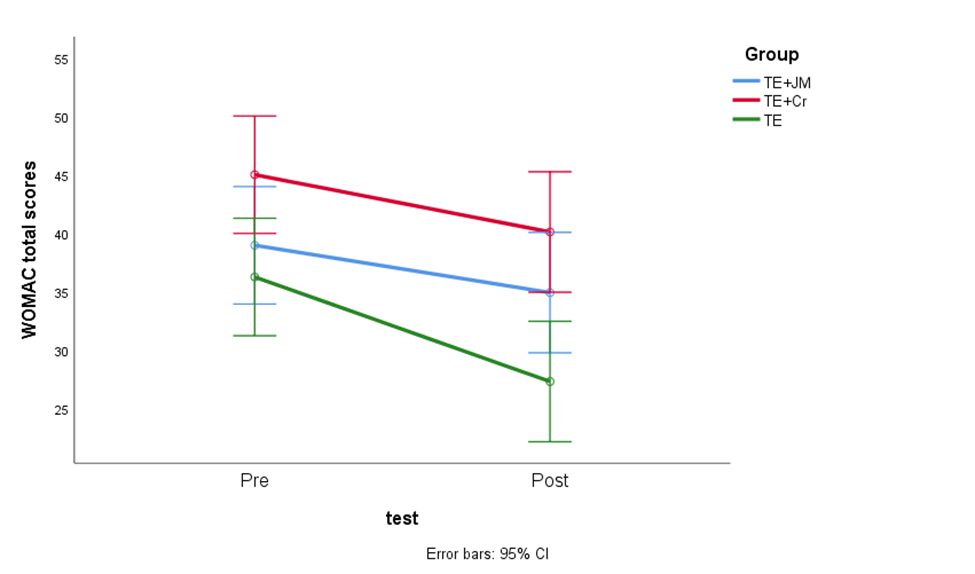


**Figure S4.** The Group x Test interaction in WOMAC total scores. Error bars represent 95% CI


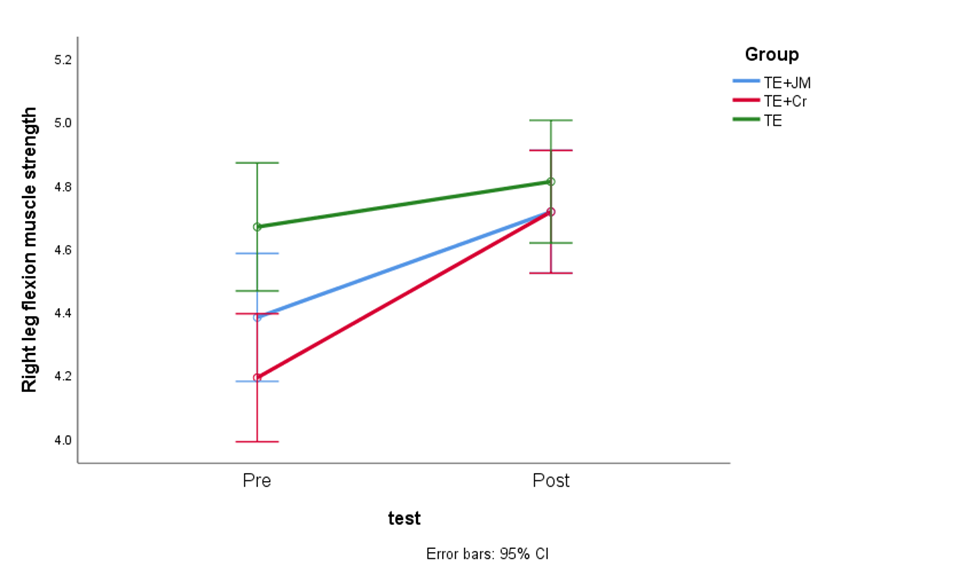
 **Figure S5.** The Group x Test interaction in right knee extension muscle strength. Error bars represent 95% CI
